# Supplementary material for: Reflective imaging improves spatiotemporal resolution and collection efficiency in light sheet microscopy
Source: Nat Commun. 2017 Nov 13;8:1452. doi: 10.1038/s41467-017-01250-8 (PMC5682293; doi:10.1038/s41467-017-01250-8)
Supplement: Supplementary file 1 — Supplementary Information [file 41467_2017_1250_MOESM1_ESM.pdf]

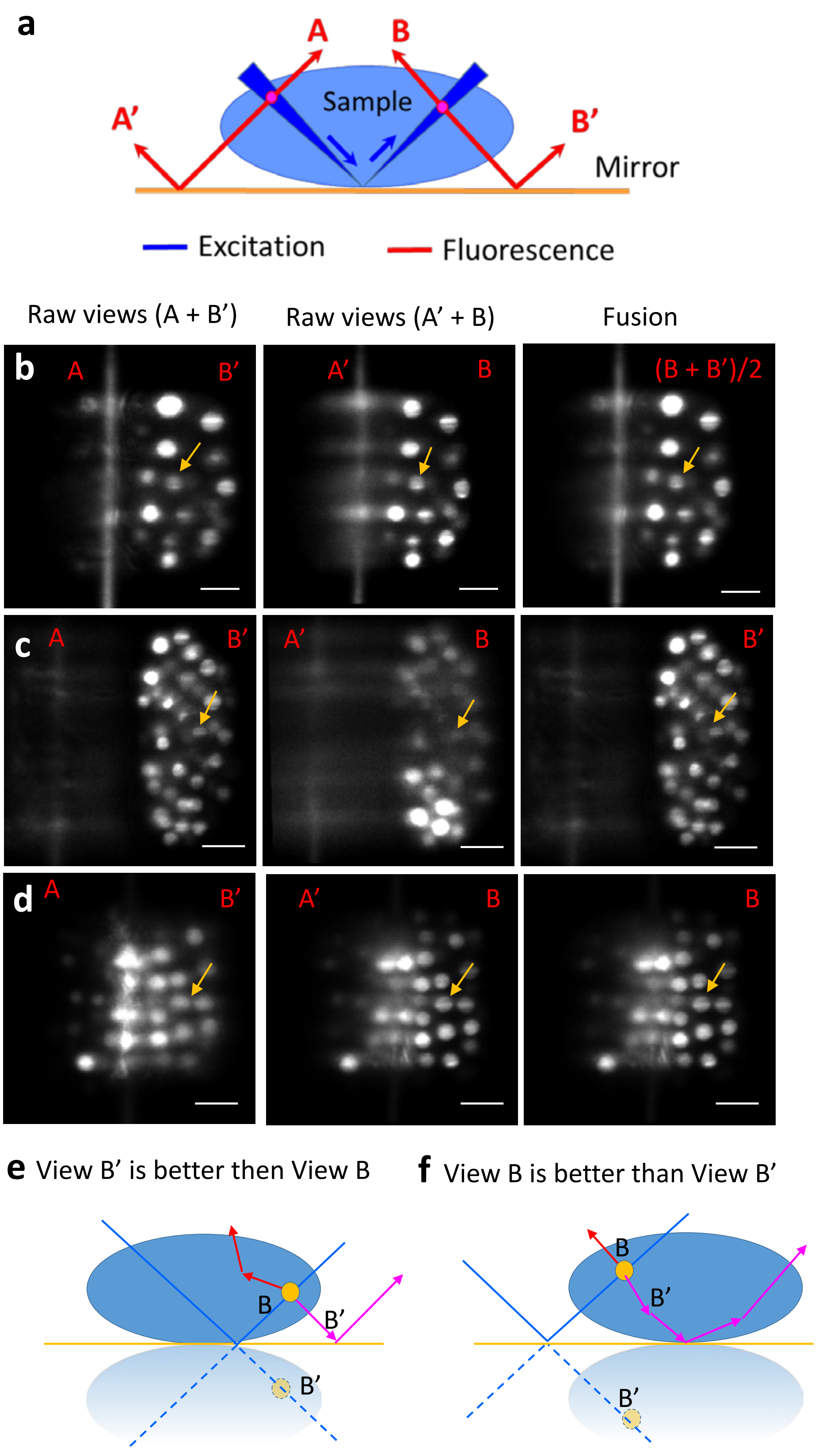

a
A
B
A’
Sample
B’
Mirror
Excitation
Fluorescence
Raw views (A + B’)
Raw views (A’ + B)
Fusion
b
A
B’
A’
B
(B + B’)/2
c
A
B’
A’
B
B’
d
A
B’
A’
B
B
e View B’ is better then View B
B
B’
B’
f View B is better than View B’
B
B’
B’

Supplementary Fig. 1, Examples of data-fusion strategy for combining normal views (e.g., B view) and mirror views (e.g., B' view) before joint deconvolution. a) Schematic higher magnification view of the specimen in Fig. 1A, showing the four views - A, A', B and B'. **b)** Averaging the two views if B and B' have similar quality. **c)** Selecting B' if its quality is better than B, for example, when the direct B view experiences more scattering as illustrated in **e)**. **d)** Selecting B if it is better than B', for example, when the mirror view B' suffers more scattering as shown in **f)**. Arrows in a-c highlight nuclei appearance using different fusion schemes. These fusion methods are also applied to A and A'. Image quality was qualitatively assessed to determine which fusion to choose, e.g in **Fig. 1, Supplementary Movie 2**. Scale bars: 10 μm.

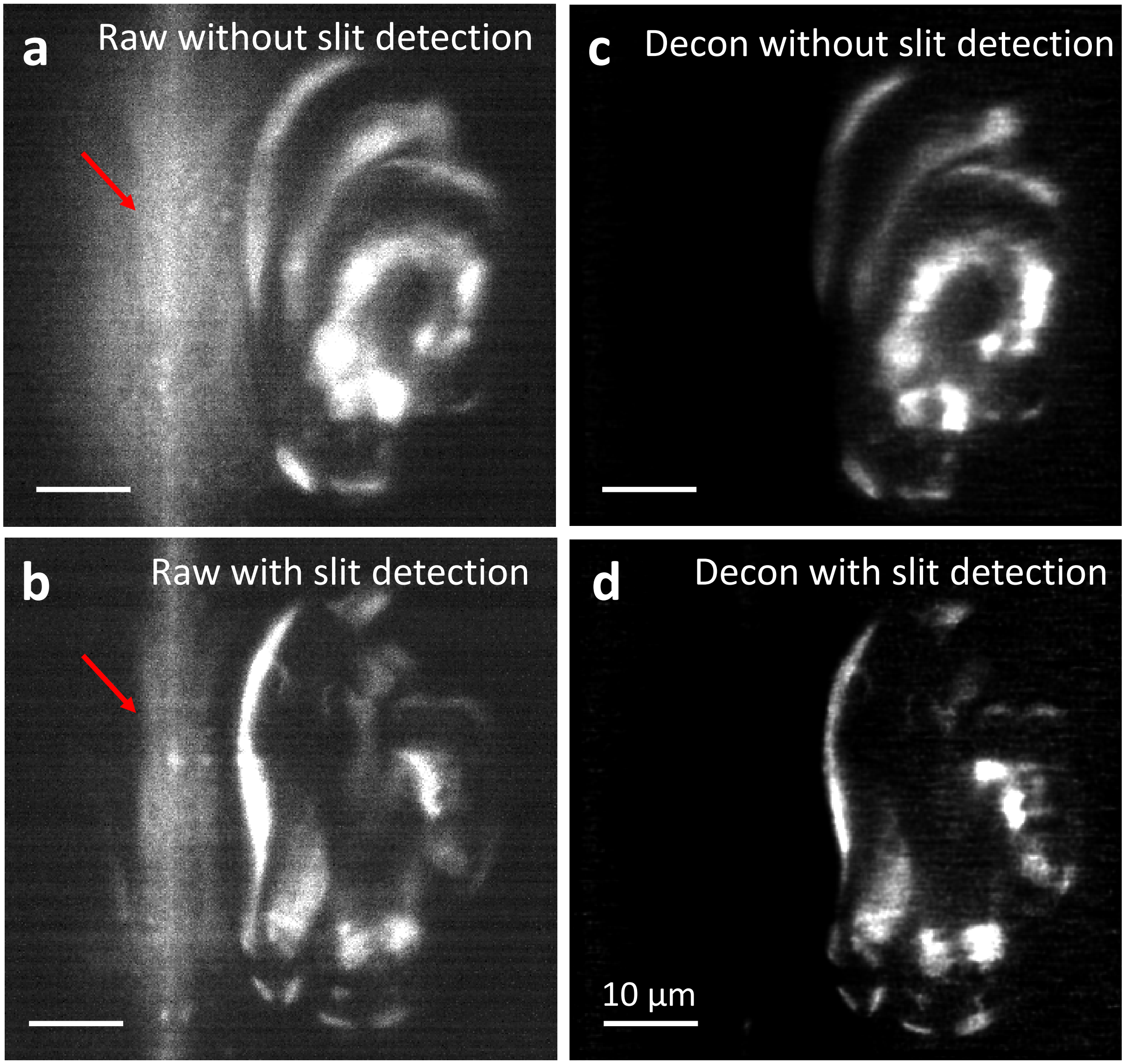

Supplementary Fig. 2, Rolling shutter slit reduces epifluorescence contamination. Example raw single planes without (**a**), and with (**b**) rolling shutter, and after deconvolution (**c, d**). Epifluorescence contamination (red arrow) is reduced after application of the rolling shutter, but deconvolution produces similar results regardless of whether a rolling shutter is used. See also **Supplementary Movies 3-4**.

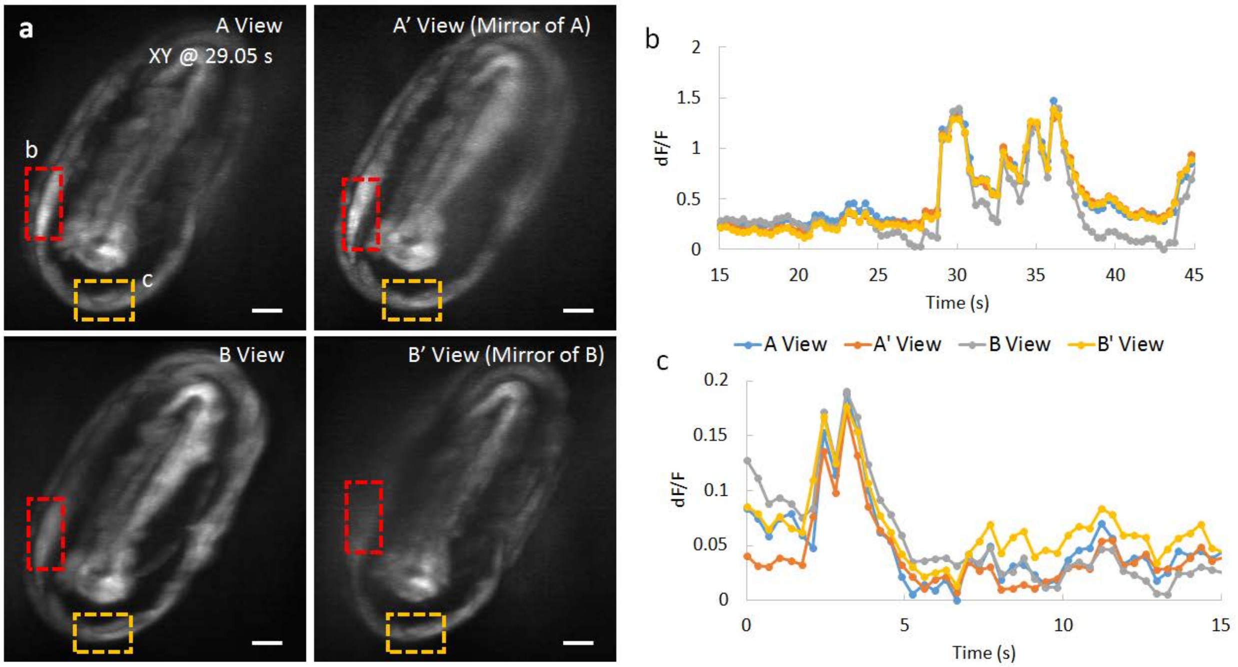

Supplementary Fig. 3, Intensity traces from raw views confirm calcium transients in *C. elegans* embryos. **a)** Representative maximum intensity projections from raw data, shown from the perspective of the coverslip. Note that planes within 1.5 µm of the coverslip were omitted from the projections, as they are badly contaminated by epifluorescence signal. **b, c)** indicate dF/F derived from red and yellow rectangular dashed regions in **a**). Note the close correspondence between intensities from each view. Scale bars: 5 µm.

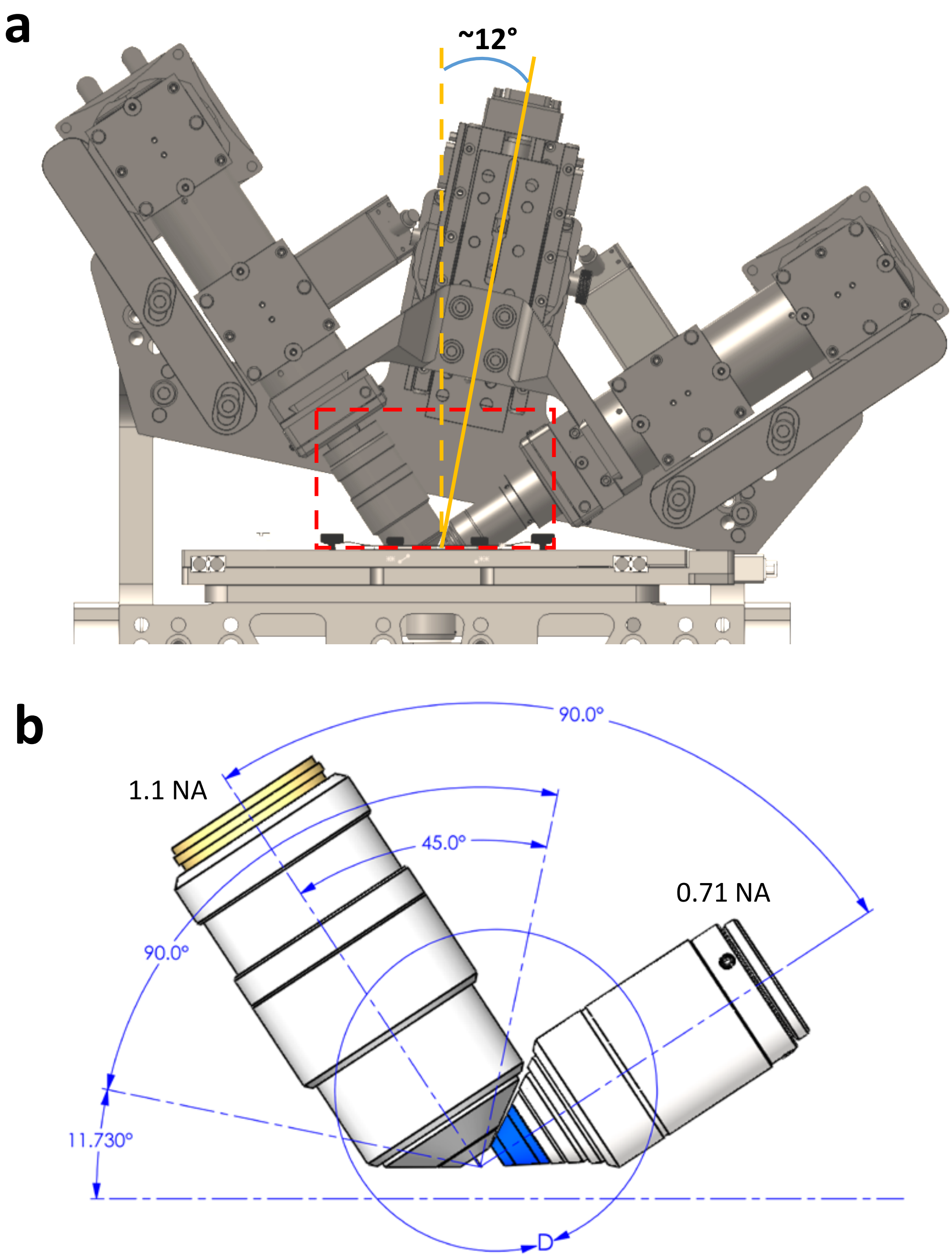

Supplementary Fig. 4, Schematic and geometry (to scale) of asymmetric 1.1/0.71 NA diSPIM system. **a)** Schematic of the diSPIM, assembled on a tilted translation stage (~12° tilt relative to the vertical axis) due to the large angular aperture (>90°) of the 1.1 NA lens. **b)** Higher magnification view of the 1.1 NA and 0.7 NA objectives.

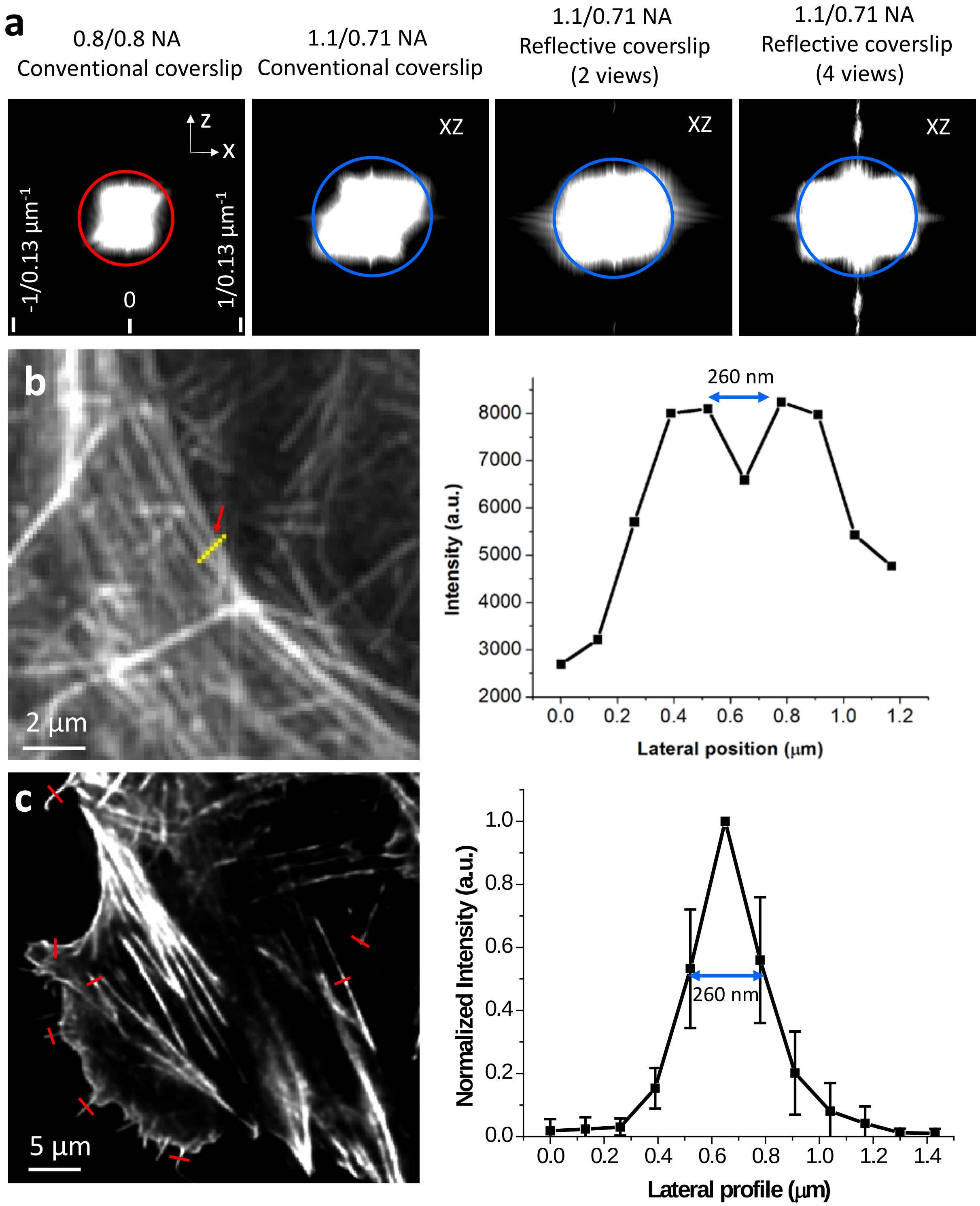

Supplementary Fig. 5, Resolution estimates derived from experiments. (a) kx/kz OTF cross sections derived from immunolabeled microtubules (presented in **Fig. 4d**) imaged with symmetric 0.8/0.8 NA diSPIM imaging on glass coverslips, 1.1/0.71 NA asymmetric diSPIM imaging on glass coverslips, dual-

view 1.1/0.71 NA diSPIM imaging on reflective coverslips, and quad-view 1.1/0.71 NA diSPIM imaging on reflective coverslips, respectively. The red and blue circles indicate the diffraction limit, 1/0.33 μm^{-1} and 1/0.26 μm^{-1}, respectively. **(b)** A higher magnification view of phalloidin labeled actin in **Fig. 4h**, imaged with 1.1/0.71 NA diSPIM on glass coverslips. The yellow line profile indicates ~260 nm separation of two actin fibers. **(c)** A higher magnification view of cellular actin in **Fig. 4j**, imaged with 1.1/0.71 NA diSPIM imaging on reflective coverslips. The right-hand curve shows an averaged intensity profile (with a FWHM of ~260 nm) over 8 actin fibers indicated by red lines in the left-hand image. Error bars represent the standard deviations.

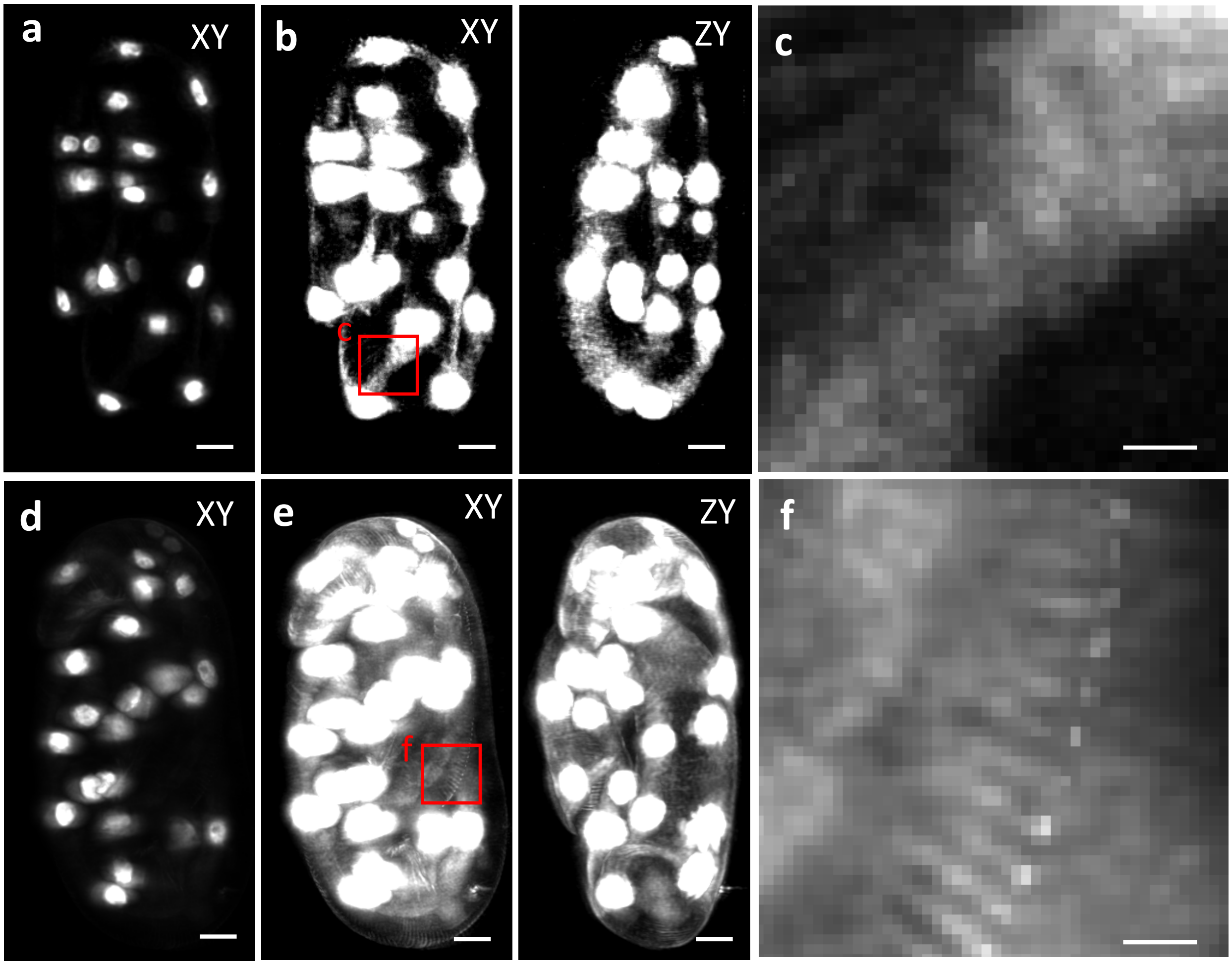

Supplementary Fig. 6, Comparisons between symmetric 0.8/0.8 NA diSPIM (a, b, c) and asymmetric 1.1/0.71 diSPIM (d, e, f) imaging when viewing similarly staged nematode embryos deposited on glass coverslips. The *C. elegans* strain is SLS-5 [*ujIS13* [*pie-1::*mCherry*::H2B + unc-119(+); pnhr-2::*mCHerry*::histone + unc-119(+)*]*II*, *wIS51* [*pSCM::*GFP *+ unc-119(+)*], *mcIS50* [*lin-26p::vab-10 (actin binding domain)::GFP + myo-2p::*GFP *+ pBluescript*]], marking seam cell nuclei and the worm surface. **a, b)** and **d**, **e)** show XY and ZY maximum intensity projection acquired with the 0.8/0.8 NA and 1.1/0.71 NA diSPIM systems respectively, note that contrast has been increased in **b, e** to better highlight dim features; **c)** and **f)** are higher magnification views of red rectangular regions in **b)** and **e)**, respectively. The higher resolution of the asymmetric diSPIM better resolves fine grooved structures labeled with the surface marker that are otherwise blurred with the symmetric diSPIM. Scale bar: 5 µm in **a, b, d, e**; 1 µm in **c**, and **f**.

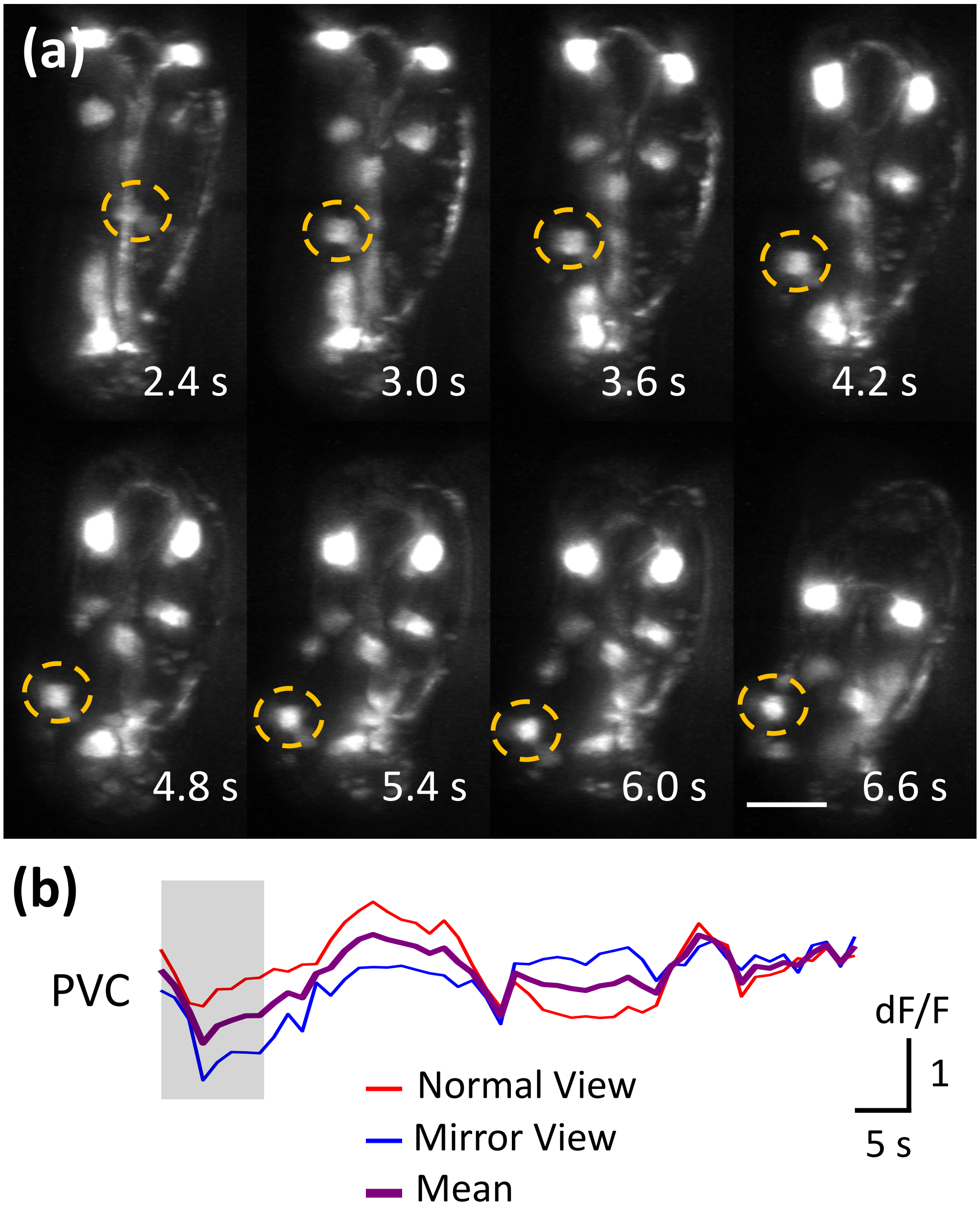

Supplementary Fig. 7, PVC dynamics during backwards movement. **a)** Selected consecutive maximum intensity projections from same dataset as in **Fig. 5d**, highlighting PVC cell (encircled with orange dashed lines). **b)** Intensity was extracted as in **Fig. 5i**, grey rectangle highlights the same temporal interval as in **Fig. 5i**, corresponding to backwards motion of animal. Scale bar: 10 µm.

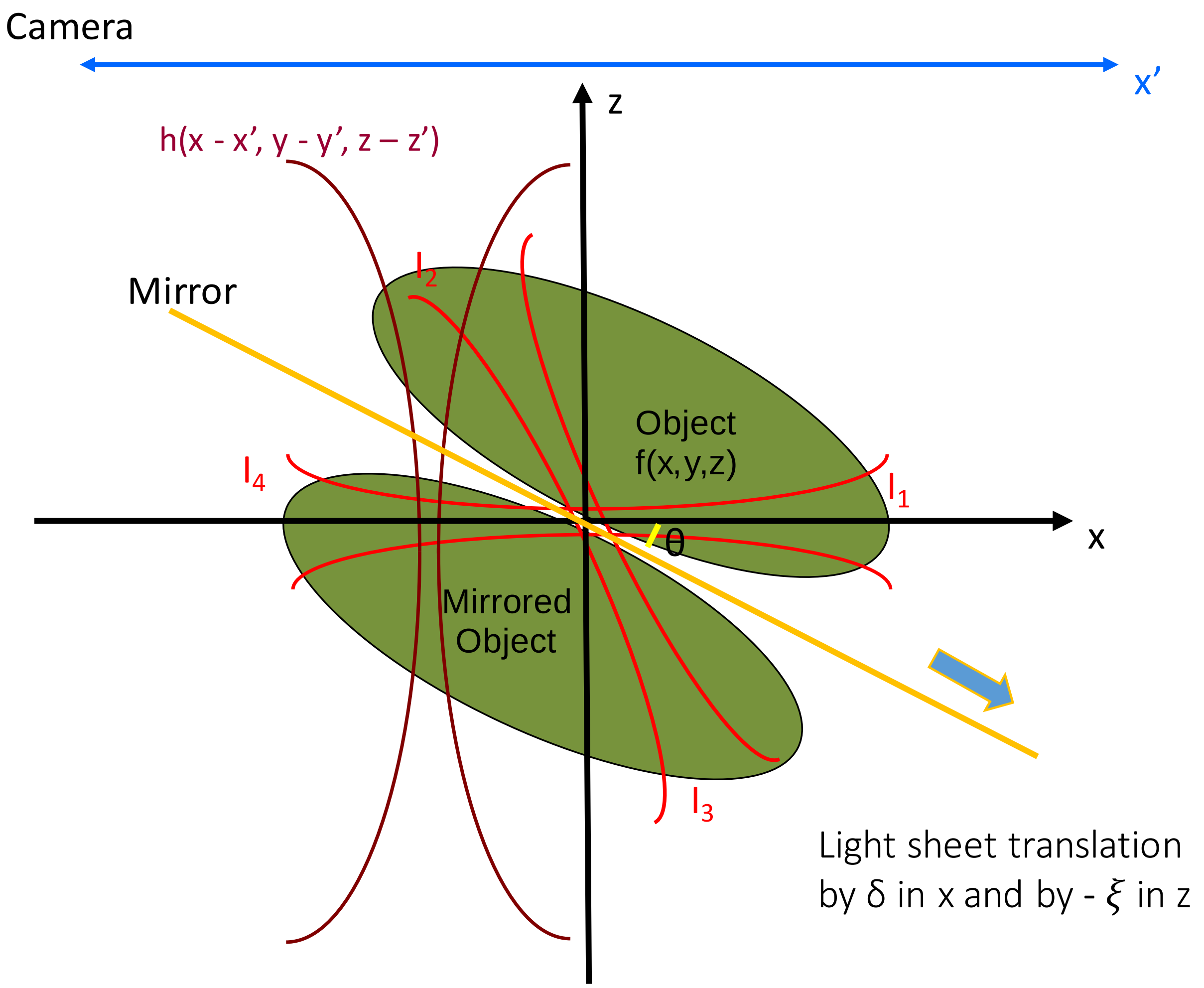

Supplementary Fig. 8, Geometry of reflective imaging, where the mirror is tilted by angle θ relative to a detection camera. In stage scanning mode, we assume that the sample translates along the mirror by δ in the x dimension and –ξ in z dimension, so that $\tan \theta = \xi/\delta$. I_1 is the stationary illumination light sheet with waist focused onto the mirror, and is perpendicular to the focal plane imaged onto the detection camera. I_2 is the reflected light sheet caused by the mirror, I_4 its mirror image, and I_3 the mirror image of I_1.

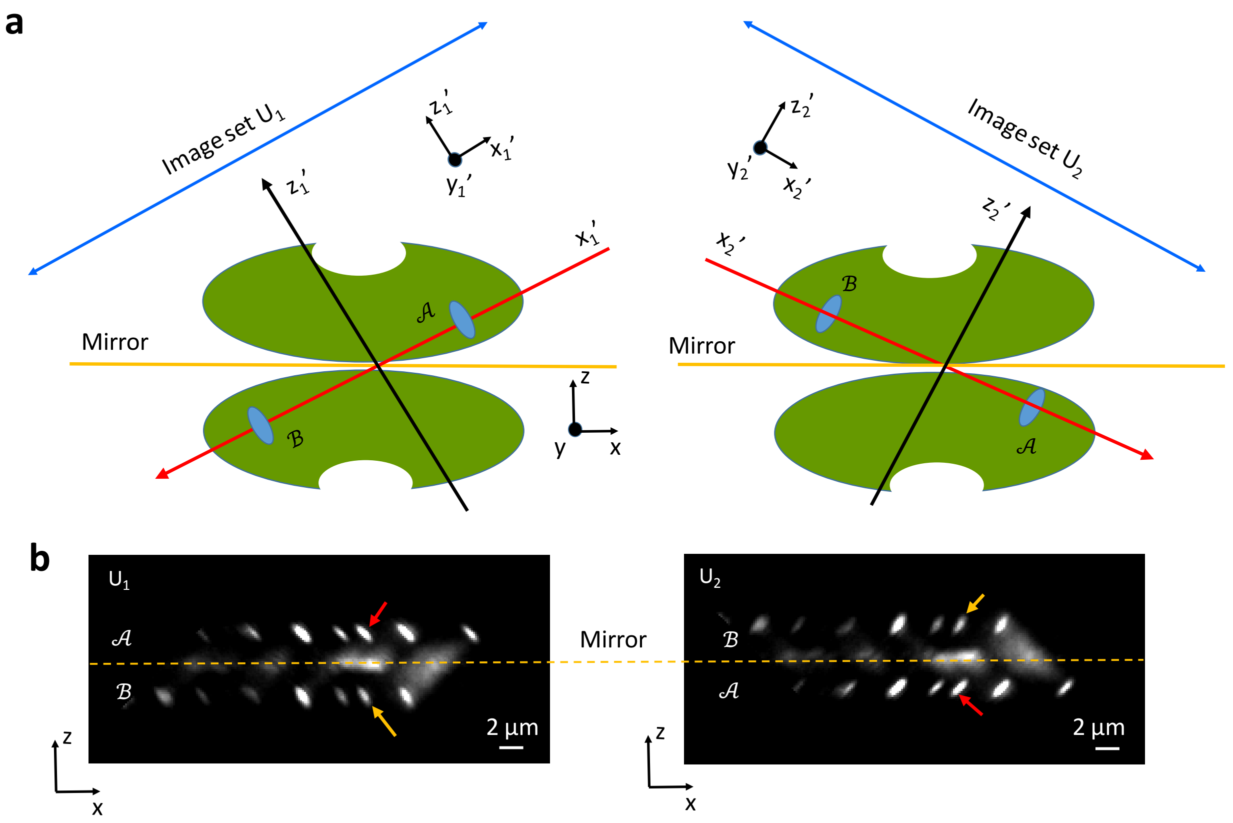

Supplementary Fig. 9, Imaging geometries used in joint deconvolution. **(a)** Schematic geometry showing composition of images U_1 and U_2, used in joint deconvolution. U_2 ($\mathcal{B} + \mathcal{A}$) is the reflection of U_1 ($\mathcal{A} + \mathcal{B}$), so that the joint deconvolution of U_1 and U_2 can be performed between $\mathcal{A}$ and $\mathcal{B}$ on the top of mirror and also between $\mathcal{B}$ and $\mathcal{A}$ beneath the mirror. As shown, $\mathcal{A}$ and $\mathcal{B}$ have complementary point spread functions; joint deconvolution recovers the best spatial resolution from each PSF and simultaneously removes epifluorescence contamination at the same time. As joint deconvolution converges, the images above or below the mirror become identical, and either is chosen for the final image. **(b)** 100 nm yellow-green fluorescence beads imaged with reflective 0.8/0.8 NA diSPIM. Both U_1 and U_2 are visualized from the perspective of the coverslip. Beads exhibit perpendicular point spread functions as highlighted by the red and orange arrows. This complementary information ensures the recovery of a more isotropic PSF after joint deconvolution between U_1 and U_2. Coordinate systems are shown defined from the perspective of each objective (x_1', y_1', and z_1' from the left-hand view; or x_2', y_2', and z_2' from the right-hand view), and that of the coverslip (x, y, and z).

Supplementary Table 1. Comparisons between symmetric diSPIM imaging on glass coverslips, symmetric diSPIM on reflective coverslips, asymmetric diSPIM on conventional coverslips, dual-view asymmetric diSPIM on reflective coverslips, and quad-view asymmetric diSPIM on reflective coverslips.

	**0.8/0.8 NA diSPIM conventional imaging**	**0.8/0.8 NA diSPIM reflective imaging**	**1.1/0.71 NA diSPIM conventional imaging**	**1.1/0.71 NA diSPIM reflective imaging**	**1.1/0.71 NA diSPIM reflective imaging**
**Geometry**	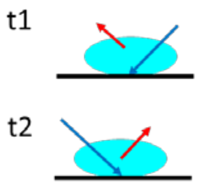	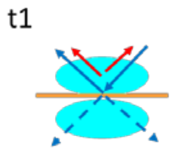	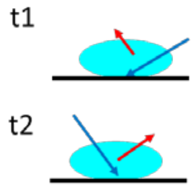	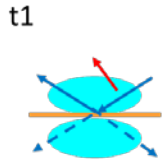	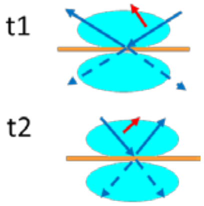
**Detection NA**	t1: 0.8 NA<br>t2: 0.8 NA	t1: all 0.8 NA	t1: 1.1 NA<br>t2: 0.71 NA	t1: both 1.1 NA	t1: both 1.1 NA<br>t2: both 0.71 NA
**Views**	2 views<br>crossed at 90°	4 views<br>crossed at 90°	2 views<br>crossed at 90°	2 views<br>crossed at 112°	4 views<br>1.1 NA views at 112°;<br>0.7 NA views at 68°.
**Solid angle**	0.8 π	1.6 π	1.2 π	1.8 π	2.4 π
**Collection efficiency**	20%	40%	30%	44%	60%
**Relative dose**	1 x	1 x	1 x	1 x	2 x
**Relative speedup**	1 x	2 x	1 x	2 x	1 x
**Resolution from the perspective of objective (X'Y'Z')**	0.33 x 0.33 x 0.33 µm^3	0.33 x 0.33 x 0.33 µm^3	0.26 x 0.26 x 0.37 µm^3	0.26 x 0.26 x 0.30 µm^3	0.26 x 0.26 x 0.28 µm^3
**Volumetric resolution enhancement**	1 x	1 x	1.44 x	1.77 x	1.90 x
**Relative OTF volume size**	48%	48%	54%	79%	100%
**Deconvolution time (360 x 360 x 360 pixels)**	0.25 s per iteration	90 s per iteration	0.25 s per iteration	90 s per iteration	180 s per iteration
**Total processing time for each figure and movie**	Fig. 4d: 5 seconds<br>Supp. Fig. 6:<br>10 seconds	Fig. 2/Movie 3:<br>39 hours<br>Movie 4: 80 hours<br>Fig. 3/Movie 5:<br>70 hours	Fig. 4d: 13 seconds<br>Fig. 4f: 40 seconds<br>Supp. Fig. 6:<br>25 seconds	Fig. 4d: 1.5 hours<br>Fig. 4g: 4.5 hours<br>Fig. 5a/Movie 9:<br>40 hours<br>Fig. 5b/Movie 10:<br>67 hours<br>Movie 11: 33 hours<br>Fig. 5d/Movie 12:<br>110 hours	Fig. 4d: 3 hours

Supplementary Table 2. Comparisons among reflective light sheet imaging, IsoView light sheet imaging and lattice light sheet imaging, as defined by various imaging metrics. Note that sizes and speeds selected correspond to the data entries noted in the 'Volumetric imaging period' row.

	**Reflective diSPIM imaging (0.8/0.8 NA)**	**Reflective light sheet imaging (0.71/1.1 NA)**	**IsoView light sheet imaging**	**Lattice light sheet imaging**
**Numerical aperture of detection lens**	0.8	1.1	0.714	1.1
**Number of Objective lenses**	2	2	4	2
**Number of detection cameras**	2	1	4	1
**Collection efficiency**	40%	44%	31%	22%
**Spatial resolution**	0.33 x 0.33 x 0.33 µm^3	0.26 x 0.26 x 0.30 µm^3	0.41x 0.42 x 0.45 µm^3	0.23 x 0.23 x 0.37 µm^3
**Volumetric imaging period (single color)**	Movie 3:<br>250 ms for four views (50 slices per view, 5 ms per slice)	Movie 12:<br>540 ms for two views (90 slices per view, 6 ms per slice)	Movie 4 (Mode 1, sequential acquisition):<br>486 ms for four views (35 slices per view, 4.6 ms per slice)<br><br>Movie 2 (Mode 2, simultaneous acquisition):<br>818 ms for four views (38 slices, 18 ms per slice)	Movie S7:<br>500 ms for single view (100 slices per view, 5 ms per slice)
**Volumetric temporal resolution (imaging + inter-imaging time)**	2. 86 Hz<br>(0.25 + 0.1 s)	1.56 Hz<br>(0.54 + 0.1 s)	Mode 1: 2 Hz<br>(0.49 + 0.01 s)<br><br>Mode 2: 1 Hz<br>(0.82 + 0.18 s)	1.54 Hz<br>(0.5 + 0.15 s)
**Volume size**	~40 x 60 x 40 µm^3	~35 x 55 x 35 µm^3	~200 x 600 x 200 µm^3	~28 x 20 x 15 µm^3
**Samples**	Cells and nematode embryos	Cells and nematode embryos	Larval zebrafish, *Drosophila* larvae and embryos	Cells, embryos in *Caenorhabditis elegans* and *Drosophila*

Supplementary Table 3. Acquisition parameters for all data used in this work. We also include the number of iterations used for deconvolving each dataset.

Sample	Figures /Movies	diSPIM system	Coverslip	Frame acquisition, ms	# of planes	Stage scanning Step, µm	Interval between volumes, s	Volume #	Iteration #
Embryonic nuclei	Fig.1	0.8/0.8 NA	Reflective Coverslip	10	100	0.69	X	1	20
Embryonic calcium (*myo3*p::GCaMP3)	Fig.2 Movie 3	0.8/0.8 NA	Reflective Coverslip	5	50	1.15	0.35	150	20
	Movie 4	0.8/0.8 NA	Reflective Coverslip	8	60	1.15	0.6	600	20
Live U2OS cell (mitochondria and membrane)	Fig. 3 Movie 5	0.8/0.8 NA	Reflective Coverslip	5 per color	80 per color	0.92	5	300	20
Fixed U2OS cell (microtubules)	Fig. 4d (column 1)	0.8/0.8 NA	GlassCove rslip	5	100	0.92	X	1	20
	Fig. 4d (column 2)	1.1/0.71 NA	Glass Coverslip	10	150	0.72	X	1	50
	Fig. 4d (column 3)	1.1/1.1 NA	Reflective Coverslip	10	150	0.72	X	1	50
	Fig. 4d (column 4)	1.1/1.1/0.71 /0.71 NA	Reflective Coverslip	10	150	0.72	X	1	100
Fixed U2OS cell (actin)	Fig. 4f Movie 7	1.1/0.71 NA	Glass Coverslip	10	180	0.72	X	1	50
	Fig. 4g Movie 8	1.1/1.1 NA	Reflective Coverslip	10	180	0.72	X	1	50
Live Jurkat T Cell (microtubules)	Fig. 5a Movie 9	1.1/1.1 NA	Reflective Coverslip	8	100	0.72	4	100	30
Live U2OS Cell (mitochondria)	Fig. 5b Movie 10	1.1/1.1 NA	Reflective Coverslip	10	80	0.95	15	200	40
Live U2OS Cell (Golgi)	Movie 11	1.1/1.1 NA	Reflective Coverslip	10	60	0.95	15	180	40
Embryonic calcium (*nmr1*p::GCaMP3)	Fig. 5d Movie 12	1.1/1.1 NA	Reflective Coverslip	6	90	0.95	0.64	50	20
Embryonic seam cells and worm surface marker	S. Fig. 6a-c	0.8/0.8 NA	Glass Coverslip	5	80	0.92	X	1	20
	S. Fig. 6d-f	1.1/0.71 NA	Glass Coverslip	10	100	0.72	X	1	20

Supplementary Note 1

1. Image formation model with reflective imaging

The following describes how we deconvolve the raw volumetric views to remove epifluorescence contamination and recover spatial resolution. We first describe our model for image formation in a symmetric diSPIM configuration, later generalizing our procedure to the asymmetric case. Furthermore, we find it helpful to consider the illumination geometry in a light-sheet scanning mode, i.e. as though we were moving the light sheets and their reflections along the mirror and through the stationary sample (even though in reality we move the sample along the mirror through stationary light sheets). The geometry and coordinate system in our symmetric diSPIM configuration are shown in **Supplementary Fig.10**. Consider a fixed coordinate system x, y, z and represent the object fluorophore distribution as $f(x, y, z)$. The z and x axes point at objectives A and B, respectively, and the mirror lies in the plane $z = -x$. We represent the effect of the mirrored coverslip simply by reflecting the object $f(x, y, z)$ across the coverslip to obtain an extended object fluorophore distribution:

$$\tilde{f}\,(x, y, z) = \begin{cases} f(x, y, z) & z > -x \\ f(-z, y, -x) & z < -x \end{cases}. \tag{1}$$

The illumination pattern is denoted $I(x, y, z)$. In general, it is the sum of the incident Gaussian beam, its direct reflection, and the mirror images of these two beams. Since the beam waist of the incident beam is focused on the reflective coverslip, this effective illumination pattern is equivalent to two crossed Gaussian beams:

$$I(x, y, z) = G\big(x, w(z)\big) + G\big(z, w(x)\big), \tag{2}$$

where

$$G\big(x, w(z)\big) = \left(\frac{w_0}{w(z)}\right)^2 \exp\left[-\frac{2\,x^2}{w^2(z)}\right], \tag{3}$$

with

$$w(z) = w_0\,\sqrt{1 + \left(\frac{z}{z_R}\right)^2}. \tag{4}$$

Here w_0 is the beam waist and z_R the Rayleigh length given by $\pi w_0{}^2/\lambda$, where λ is the wavelength. A similar expression holds for $G\big(z, w(x)\big)$ with the x and z variables reversed.

Note that there is no y dependence since these light beams are scanned (constant) in the y direction to create the light sheet.

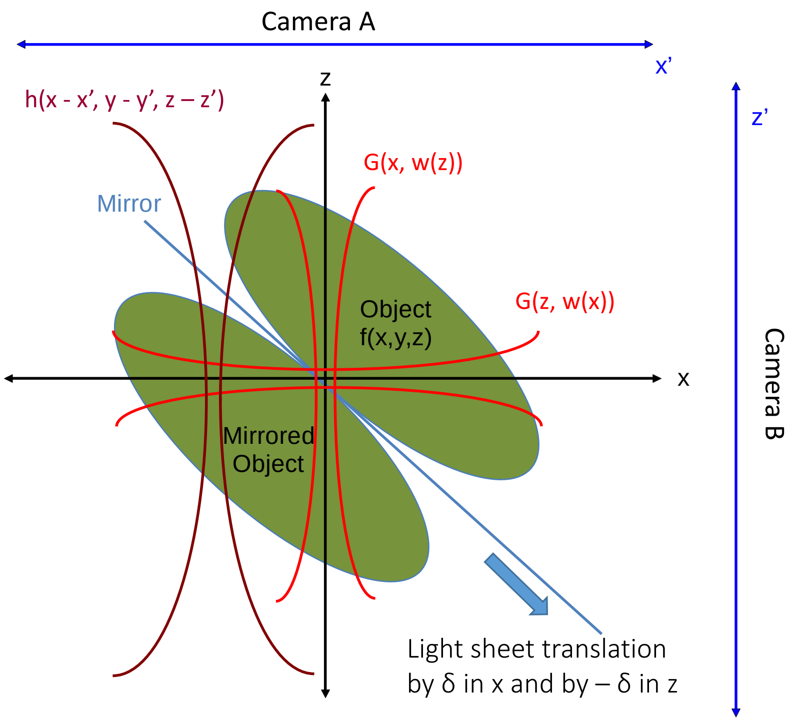

***Supplementary Fig. 10**, Geometry for reflective imaging in symmetric diSPIM configuration.*

We assume the excitation pattern is moved a distance $\sqrt{2}\delta$ corresponding to a positive increment of δ in the x direction and a negative increment $-\delta$ in the z direction. This shift of the excitation pattern relative to the fixed coordinates can be represented as

$$I(x-\delta, y, z+\delta).$$

Consider camera A, looking down the z axis through lens A, recording an image in camera coordinates x', y'. If we assume the lens has an emission point spread function $\mathrm{h_A}(x, y, z)$ that is shift invariant in x and y focused at the plane $z = -\delta$, then the image recorded by camera A can be written

$$u^{\mathrm{A}}(x', y')|_{z'=-\delta} = \int\int\int \mathrm{dxdydz}\, \tilde{f}\,(x, y, z) I(x-\delta, y, z+\delta) h_{\mathrm{A}}(x-x', y-y', z+\delta). \qquad (5)$$

Likewise, camera B looks down the x axis through lens B. It will record an image in camera coordinates y', z'. If we assume the lens has a response function $h_{\mathrm{B}}(x, y, z)$ that is shift invariant in y and z and focused at the plane $x = \delta$, the image recorded by camera B can be written

$$u^{\mathrm{B}}(y', z')|_{x'=\delta} = \int\int\int \mathrm{dxdydz}\, \tilde{f}\,(x, y, z) I(x-\delta, y, z+\delta) h_{\mathrm{B}}(x-\delta, y-y', z-z'). \qquad (6)$$

Incrementing δ to scan the light sheet through the object produces two light-sheet scanning stacks. For example, by replacing δ with z' in Equation 5, we can describe the 3D stack acquired by camera A as:

$$u_{\mathrm{LS}}^{\mathrm{A}}(x', y', z') = \int\int\int \mathrm{dxdydz}\, \tilde{f}\,(x, y, z) I(x+z', y, z-z') h_{\mathrm{A}}(x-x', y-y', z-z'). \qquad (7)$$

This light-sheet scanning stack can be decomposed into two terms if we substitute the illumination function described in Equations 2-4:

$$
\begin{aligned}
u_{\mathrm{LS}}^{\mathrm{A}}(x',y',z') \quad = \quad & \int\int\int \mathrm{d}x\mathrm{d}y\mathrm{d}z\, \tilde{f}\,(x,y,z)G(z-z',w(x+z'))h_{\mathrm{A}}(x-x',y-y',z-z') \\
& +\int\int\int \mathrm{d}x\mathrm{d}y\mathrm{d}z\, \tilde{f}\,(x,y,z)G(x+z',w(z-z'))h_{\mathrm{A}}(x-x',y-y',z-z')
\end{aligned}. \quad (8)
$$

The first term represents conventional in-focus imaging of the light sheet that is parallel to objective A. The second term represents epifluorescence contamination. This can be seen by considering that in the absence of the mirror, when objective A is acquiring data, the illumination is delivered solely from objective B along the x axis and we can drop the second term, leaving:

$$
\begin{aligned}
& u_{\mathrm{LS}}^{\mathrm{A-no-mirror}}(x',y',z') \\
& = \int\int\int \mathrm{d}x\mathrm{d}y\mathrm{d}z\, \tilde{f}\,(x,y,z)G\big(z-z',w(x+z')\big)h_{\mathrm{A}}(x-x',y-y',z-z'). \quad (9)
\end{aligned}
$$

To further explore the connection with our previous geometry, let us temporarily ignore the hourglass shape of the light sheet so that $w(x+z') \approx w_0$ is a constant (in practice in this work we account for the full hourglass excitation pattern described by Equations 2-4). Then we can define

$$
\mathrm{psf}(x-x',y-y',z-z') \equiv G(z-z',w_0)h_{\mathrm{A}}(x-x',y-y',z-z') \quad (10)
$$

and we have

$$
u_{\mathrm{LS}}^{\mathrm{A-no-mirror}}(x',y',z') = \int\int\int \mathrm{d}x\mathrm{d}y\mathrm{d}z\, \tilde{f}\,(x,y,z)\mathrm{psf}(x-x',y-y',z-z'). \quad (11)
$$

This is just a 3D convolution between the object of interest and a system PSF that is the product of the light-sheet profile and the objective lens detection PSF. Such an equation and a similar one for view B implicitly form the basis for the joint, shift-invariant deconvolution procedure employed in diSPIM[1].

Let's go back to the case with the mirror but consider the epifluorescence term under the same assumptions, ignoring the hourglass shape of the light sheet:

$$
u_{\mathrm{LS}}^{\mathrm{A-epi}}(x',y',z') = \int\int\int \mathrm{d}x\mathrm{d}y\mathrm{d}z\, \tilde{f}\,(x,y,z)G(x+z',w_0)h(x-x',\mathrm{y}-y',z-z'). \quad (12)
$$

Note that this expression is not a shift-invariant convolution between the object of interest and a single PSF. The spatially varying epifluorescence illumination destroys the shift invariance.

2. Richardson-Lucy update for shift variant PSF

So far everything has been in continuous notation. To implement Richardson-Lucy deconvolution, thus estimating the object from the images, we transition to discrete notation. Specifically it is helpful to consider each measured three-dimensional stack as a one-dimensional vector $\boldsymbol{u}$ (in practice we will not actually organize our data in that way, it just helps to think of it this way) and each desired deconvolved object distribution as a vector $\tilde{\mathbf{f}}$. Let's assume that the object has K voxels $\tilde{f}_k$, $k = 0,\ldots,\mathrm{K}-1$, and the data a total of J voxels $u_j, j = 0,\ldots,\mathrm{J}-1$. Note that J does not need to equal K because even if each data stack has the same size as the final image stack, the vector u denotes the complete set of data (i.e., the views from both objective lens A and objective lens B). Equation 7 above is linear and thus of the form

$$
\mathbf{u} = \mathrm{M}\,\tilde{\mathbf{f}}, \quad (13)
$$

where M is a matrix representing the entire mapping between object distribution and data. For a general matrix M that is not necessarily shift-invariant or normalized to 1, the Richardson-Lucy update for the current estimate $\hat{\mathbf{f}}_k^{(n)}$ of the object $\tilde{f}$ at voxel k is given by

$$\hat{\mathbf{f}}_k^{(n+1)} = \hat{\mathbf{f}}_k^{(n)} \frac{1}{v_k} \sum_{j=0}^{\mathrm{J}-1} \mathrm{M}_{jk} \frac{u_j}{\left[\mathrm{M}\, \hat{\mathbf{f}}_k^{(n)}\right]_j}, \tag{14}$$

where

$$v_k = \sum_{j=0}^{\mathrm{J}-1} \mathrm{M}_{jk} \tag{15}$$

is the kth element of a sensitivity or normalization image to ensure every iterative update is normalized. There are a few key pieces here. In $\mathrm{M}\, \hat{\mathbf{f}}_k^{(n)}$ we need to be able to apply the matrix M to our latest estimate of the data. We then divide that voxelwise into the measured data u. The resulting ratio vector $\mathbf{r}$ has elements

$$r_j \equiv \frac{u_j}{\left[\mathrm{M}\, \hat{\mathbf{f}}_k^{(n)}\right]_j} \tag{16}$$

and has the dimensions of the complete data stack. The sum

$$\sum_{j=0}^{\mathrm{J}-1} \mathrm{M}_{jk} \frac{u_j}{\left[\mathrm{M}\, \hat{\mathbf{f}}_k^{(n)}\right]_j} = \sum_{j=0}^{\mathrm{J}-1} \mathrm{M}_{jk}\, r_j = [\mathrm{M}^T \mathbf{r}]_k \tag{17}$$

is equivalent to multiplying the vector $\mathbf{r}$ by M^{T} (where T denotes transpose) and picking out the kth element. Note that from this point of view, the normalization vector $\mathbf{v} = M^T \mathbf{1}$, where $\mathbf{1}$ denotes a vector of ones.

3. Implementation of single-view Richardson-Lucy update

By changing the one-dimensional vectors to image stacks (e.g. vectors $\mathbf{u}, \mathbf{v}, \hat{\mathbf{f}}$ to images $U, V, \hat{F}$, respectively), we can rewrite the above Richardson-Lucy iterative update (Equation 18) as:

$$\hat{F}^{(n+1)} = \hat{F}^{(n)} \frac{1}{V} \left[\mathcal{M}^T \frac{U}{\mathcal{M}\hat{F}^{(n)}}\right], \tag{18}$$

where we use $\mathcal{M}$ to denote the linear imaging operator (which is not explicitly a matrix in this notation). To implement the Richardson-Lucy update, we: (1) apply $\mathcal{M}$ to an estimate of object $\hat{F}^{(n)}$ to get a data stack *E*, see **Supplementary Fig. 11a-d**; (2) divide the measured image stack U by this calculated image stack *E*, resulting in a ratio image $R = \frac{U}{\mathcal{M}\hat{F}^{(n)}}$, see **Supplementary Fig. 11e**; (3) apply $\mathcal{M}^T$ to R to get a

correction image stack C, see **Supplementary Fig. 11f-i**; (4) update the current estimate $\hat{F}^{(n)}$ by multiplying it with the correction image stack $C = \mathcal{M}^T R$ and dividing by the normalization image stack V, see **Supplementary Fig. 11j**. Here $V = \mathcal{M}^T \mathbf{1}$, where $\mathbf{1}$ denotes a unity image stack.

The key point in performing R-L is applying $\mathcal{M}$ and the transpose of $\mathcal{M}$. We now consider the more general diSPIM configuration (**Online Methods**, **Supplementary Fig. 8**) when the mirror is angled at θ relative to the detection lens (θ $= 45^\circ$ for symmetrical diSPIM), and assume the object is shifted along the mirror by an increment δ in the x direction and an increment of $-\delta\tan(\theta)$ in the z direction. From the perspective of light-sheet scanning mode (see Equation 4 in Online Methods), $\mathcal{M}$ can be broken into blocks $\mathcal{M}_\delta$ corresponding to the different light-sheet offsets δ. For each such block, image formation can be described as a 3 cascaded operations, given by

$$\mathcal{M}_\delta = \mathcal{P}\mathcal{H}\mathcal{D}_\delta \tag{19}$$

To implement this on the image stacks we loop over δ, and for each increment δ, we

1. Shift the illumination pattern by δ in x and $-\delta\tan\theta$ in z (**Supplementary Fig. 11a**), and multiply the estimate $\hat{F}^{(n)}$ by the shifted illumination function $I(x-\delta, y, z+\delta\tan\theta)$ (**Supplementary Fig. 11b**). We represent this operation by an operator D_δ.
2. Loop over z in the object and at each z, perform 2D convolution with the lens response function (**Supplementary Fig. 11c**). This operation can be represented by an operator $\mathcal{H}$.
3. Collapse over the z coordinate **(Supplementary Fig. 11d)**. This operation is represented by a projection operator $\mathcal{P}$, which sums convolved elements at each xy position along z.

The transpose of $\mathcal{M}$ can be implemented by summing over the applications of $\mathcal{M}_\delta$, each of which is equivalent to applying the three transposed operations in reverse order, i.e.,

$$\mathcal{M}_\delta^{\mathrm{T}} = \mathcal{D}_\delta^{T}\mathcal{H}^{\mathrm{T}}\mathcal{P}^{\mathrm{T}} \tag{20}$$

Again, we loop over δ, and for each δ, we

4. Perform back projection $\mathcal{P}^{\mathrm{T}}$. This operation is often used in tomography, and entails smearing the ratio image $R = \frac{U}{\mathcal{M}\hat{F}^{(n)}}$ back across the image grid along z (**Supplementary Fig. 11f**).
5. $\mathcal{H}^{\mathrm{T}}$ applies convolution with the adjoint point spread functions, i.e. 2D convolution of back projected R with the flipped PSF at each z to form a quantity Q. See **Supplementary Fig. 11g**.
6. Since D_δ is effectively a diagonal operator, $\mathcal{D}_\delta^T = \mathcal{D}_\delta$ and thus multiplies Q with the shifted illumination pattern (shift the illumination pattern by δ in x and $-\delta\tan\theta$ in z). See **Supplementary Fig. 11h**.
7. Add the resulting image stack to the accumulating correction image stack C that will multiply the latest iteration of the object stack.

Note that the Richardson-Lucy update (Equation 18) in essence is a series of 2D processes for recovering $U(x,y)$ at each δ: perform steps 1-6 above at each δ, then sum over δ to update the estimate (**Supplementary Fig. 11j**), eventually recovering an estimate of $U(x,y,z)$ with reduced epifluorescence contamination. Equivalently, the 2D convolution involved in implementing $\mathcal{P}\mathcal{H}$ in

Equation 19 can be written as the 3D convolution of $I(\mathrm{x}-\delta,\mathrm{y},\mathrm{z}+\delta\tan\theta)\,\hat{\mathrm{F}}^{(\mathrm{n})}$ with the 3D response function (PSF) of the detection lens, then picking out the xy data at the axial plane $\mathrm{z}=-\delta$; the 2D convolution when implementing $\mathcal{H}^T\mathcal{P}^T$ is equivalent to 3D convolution of the data at $z=-\delta$ (i.e., creating a 3D stack with the nonzero elements at $z=-\delta$ and zeros at other z) with the 3D PSF of the detection lens.

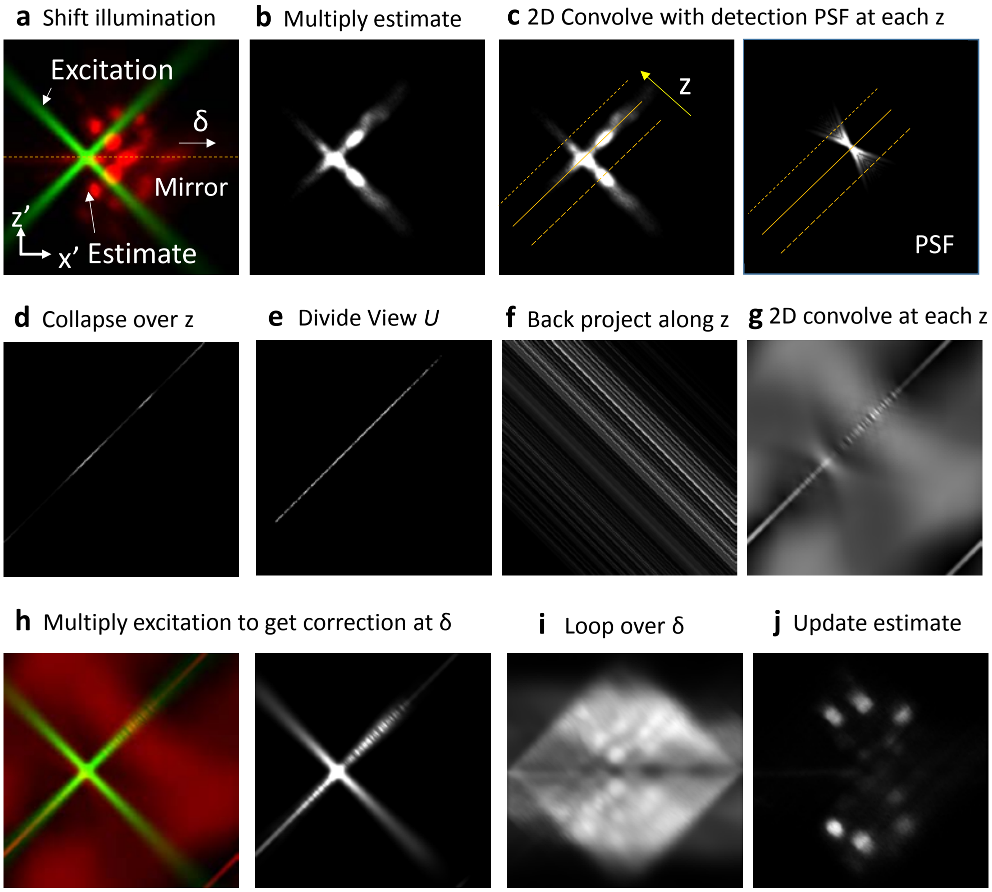

***Supplementary Fig. 11**, Schematic of one Richardson-Lucy update at iteration n in the x'-z' plane. **(a-d)** applying operator $\mathcal{M}$ to the current estimate of object $\hat{F}^{(n)}$: **a)** shifting the excitation pattern along mirror by an increment δ; **b)** multiplying the estimate $\hat{F}^{(n)}$ with shifted the excitation pattern; **c)** 2D convolving with the detection PSF at each z; **d)** projecting over z to apply the forward transformation $\mathcal{M}\,\hat{F}^{(n)}$; **(e)** dividing the measured image U by $\mathcal{M}\,\hat{F}^{(n)}$; **(f-h)** Applying transposed matrix $\mathcal{M}^T$ to the ratio image R: **f)** Projecting R back along z; **g)** Applying 2D convolution with transposed detection PSF at each z and summing along z; **h)** multiplying with mirrored excitation pattern; **i)** looping over δ to obtain a correction image C; **(j)** updating the current estimate $\hat{F}^{(n)}$ with the correction image C and normalization image V to obtain a new estimate $\hat{F}^{(n+1)}$.*

4. Implementation of dual-view Richardson-Lucy deconvolution in MATLAB

In order to combine the benefits of epifluorescence removal while obtaining a more isotropic PSF, we formed two views U_1 and U_2 as defined in the Online Methods (Section 3: Fusing raw volumetric views to create two views), then jointly deconvolved the two views according to the Online Methods (Section 4: Joint deconvolution for epifluorescence removal and resolution recovery). We implemented the procedure in MATLAB (**Supplementary Software**) using two coordinate systems, either the perspective of the objective lens for 0.8/0.8 NA reflective diSPIM imaging, or the perspective of the coverslip for 1.1/0.71 NA reflective diSPIM imaging.

When considering diSPIM in the light-sheet scanning mode, we usually define the coordinate system from one of the objective's perspectives (for example, x_1', y_1', and z_1' from the left-hand view; or x_2', y_2', and z_2' from the right-hand view, **Supplementary Fig. 9**). Here, x_1' and x_2' correspond to the propagation direction of the light sheet, y_1' and y_2' to the direction along the light sheet width (y_1' and y_2' are identical, and both are denoted as y' going forward), and z_1' and z_2' are perpendicular to the $x_1'y_1'$ plane and $x_2'y_2'$ plane, respectively. In the 0.8/0.8 NA symmetric, reflective diSPIM configuration, the two views $U_1(x_1', y', z_1')$ and $U_2(x_2', y', z_2')$ are perpendicular, i.e., x_2' is parallel to z_1', and z_2' is parallel to x_1'. Thus the transformation of the estimate $\hat{F}^{(n)}$ from one perspective to the other perspective (i.e., 90 degrees rotation from $x_1'z_1'$ to $x_2'z_2'$ along y' axis) during the joint Richardson-Lucy update can be easily implemented by switching the lateral and axial axes. Thus, we retain this coordinate system when processing the symmetric, reflective diSPIM data.

However, in the 1.1/0.71 NA asymmetric, reflective case, views $U_1(x_1', y', z_1')$ and $U_2(x_2', y', z_2')$ are crossed at 112 degrees. The non-orthogonal rotation of the estimate $\hat{F}^{(n)}$ from one view to the other view in each joint Richardson-Lucy update is inconvenient, requiring additional interpolation. Therefore, we visualize both views from the perspective of the coverslip, so that the two views (U_1 and U_2) share the same coordinate system (x, y, and z in **Supplementary Fig. 9**), and we don't need to transform between the views and estimates $\hat{F}^{(n)}$ during the joint deconvolution update. The transformation from coordinates (x_1', y', z_1') and (x_2', y', z_2') to (x, y, z) is shown in Equations 21 and 22:

$$x = x_1'\cos\theta - z_1'\sin\theta$$
$$z = x_1'\sin\theta + z_1'\cos\theta$$
$$y = y' \tag{21}$$

$$x = x_2'\cos\theta + z_2'\sin\theta$$
$$z = x_2'\sin\theta - z_2'\cos\theta$$
$$y = y'. \tag{22}$$

Here θ is defined as the angle between x_1' and the lateral direction of mirror (i.e., x).

Supplementary Note 2

1. Modelling PSF without rolling shutter slit mode

Without rolling-shutter slit detection, the point spread function h(x,y,z) used in implementing operator $\mathcal{H}$ in the convolution process (in Equations 19 and 20) is given by the widefield PSF of the detection objective. We simulated widefield PSFs with the freely available PSF Generator (ImageJ plugin, http://bigwww.epfl.ch/algorithms/psfgenerator/ using the 'Born and Wolf' model with appropriate numerical aperture, i.e. 0.8/0.8 NA crossed at 90 degrees for reflective, symmetric diSPIM imaging, 1.1/1.1 NA crossed at 112 degrees for reflective, asymmetric diSPIM imaging, 0.71/0.71 NA crossed at 68 degrees for quadruple-view reflective light sheet imaging, refractive index of immersion medium 1.33; wavelength 525 nm as is typical for GFP).

2. Modelling PSF with rolling shutter slit mode

When using the rolling-shutter slit mode, we consider a 2D model for notational simplicity since the rolling direction is along the y axis.

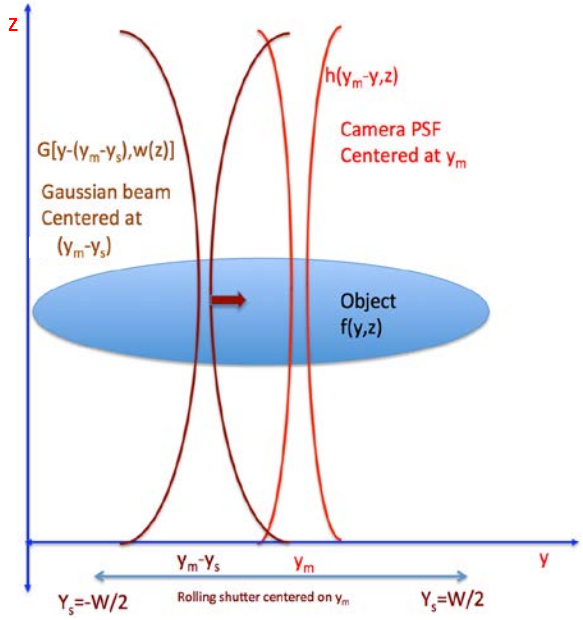

Supplementary Fig. 12, *Geometry for the rolling shutter.*

In **Supplementary Fig. 12**, we show the y-z plane, with the Gaussian beam that is swept in y to create the light sheet shown in brown and the response function of the pixel at y_m (m for measured) shown in red. The illumination Gaussian beam *G* is shown centered at $y_m - y_s$, where s stands for shift. The object fluorophore distribution is denoted $f(y,z)$. The widefield detection PSF is denoted as *h*. The rolling shutter can be accounted for by constraining the measurement to only be responsive to shifts of the Gaussian beam between $-\mathrm{W}/2$ and $\mathrm{W}/2$, where W is the slit width. The measurement at y_m will be given by

$$m(y_m, z) = \int_{-\mathrm{W}/2}^{\mathrm{W}/2} \mathrm{d}y_s \int_{-\infty}^{\infty} \mathrm{d}y\, h(y_m - y, z) G[y - (y_m - y_s), w(z)] f(y, z). \quad (23)$$

We can swap the order of the integrals and rearrange terms to obtain

$$m(y_m, z) = \int_{-\infty}^{\infty} \mathrm{d}y\, f(y,z) h(y_m - y, z) \int_{-\mathrm{W}/2}^{\mathrm{W}/2} \mathrm{d}y_s\, G[y - (y_m - y_s), w(z)]. \tag{24}$$

The Gaussian beam is given by

$$G(y, w(z)) = \left(\frac{w_0}{w(z)}\right)^2 \exp[-2y^2/w^2(z)]. \tag{25}$$

So we have to evaluate an integral of the form

$$\int_{-\mathrm{W}/2}^{\mathrm{W}/2} \mathrm{d}y_s\, G[(y - y_m) + y_s, w(z)] = \left(\frac{w_0}{w(z)}\right)^2 \int_{-\mathrm{W}/2}^{\mathrm{W}/2} \mathrm{d}y_s \exp\left[-2\left((y - y_m) + y_s\right)^2 / w^2(z)\right]. \tag{26}$$

This has the form

$$\int_{-\mathrm{W}/2}^{\mathrm{W}/2} \mathrm{d}y_s\, e^{\left[-\frac{(a+y_s)^2}{b^2}\right]}, \tag{27}$$

where $a = y - y_m$ and $b = \frac{w(z)}{\sqrt{2}}$. The integral is given by

$$\int_{-\mathrm{W}/2}^{\mathrm{W}/2} \mathrm{d}y_s\, e^{\left[-\frac{(a+y_s)^2}{b^2}\right]} = \frac{1}{2}\sqrt{\pi} b \left[\mathrm{erf}\left(\frac{a + \frac{\mathrm{W}}{2}}{b}\right) - \mathrm{erf}\left(\frac{a - \frac{\mathrm{W}}{2}}{b}\right)\right], \tag{28}$$

where

$$\mathrm{erf}(x) = \frac{2}{\sqrt{\pi}} \int_0^x dt\, e^{-t^2}. \tag{29}$$

So

$$\int_{-\mathrm{W}/2}^{\mathrm{W}/2} \mathrm{d}y_s\, G[(y - y_m) + y_s, w(z)] = \left(\frac{w_0}{w(z)}\right)^2 \frac{1}{2}\sqrt{\pi} \frac{w(z)}{\sqrt{2}} \left[\mathrm{erf}\left(\frac{(y-y_m)+\frac{\mathrm{W}}{2}}{\frac{w(z)}{\sqrt{2}}}\right) - \mathrm{erf}\left(\frac{(y-y_m)-\frac{\mathrm{W}}{2}}{\frac{w(z)}{\sqrt{2}}}\right)\right]. \tag{30}$$

Note that in Equation 24, the inner integral multiplies the wide-field PSF $h(y_m - y, z)$ and also depends only on $(y - y_m)$. So we can model the effect of the rolling shutter by leaving the original illumination function with no y dependence in place, but modifying a generic PSF $h(y, z)$ by multiplying it with a slit function

$$s(y, z) = \left(\frac{w_0}{w(z)}\right)^2 \frac{1}{2}\sqrt{\pi} \frac{w(z)}{\sqrt{2}} \left[\mathrm{erf}\left(\frac{y + \left(\frac{\mathrm{W}}{2}\right)}{\frac{w(z)}{\sqrt{2}}}\right) - \mathrm{erf}\left(\frac{y - \left(\frac{\mathrm{W}}{2}\right)}{\frac{w(z)}{\sqrt{2}}}\right)\right]. \tag{31}$$

Note that we have replaced $y - y_m$ by y to represent the generic shift invariant PSF that we will use at all y_m. Now the point spread function contained in $\mathcal{H}$ in the 2D convolution process in Equations 19-20 (**Supplementary Note 1**) under the rolling shutter slit mode can be simply expressed as:

$$h_{new}(x, y, z) = h(x, y, z) s(y, z). \tag{32}$$

Supplementary Note 3

The reflective light sheet imaging could in principle be implemented with only a single high-NA objective for introducing direct and reflected light sheets and collecting the corresponding views, thus simplifying instrument design relative to the two-objective design presented in this paper (**Supplementary Fig. 13a**). This simplified configuration allows the simultaneous acquisition of the reflected light sheet view, B, and the mirror image of the direct light sheet view, A'. Again, by treating B and A' as a single image U_1 and forming the reflected image U_2, we can jointly deconvolve the two images to remove epifluorescence contamination and improve spatiotemporal resolution, according to Equations 11 in Online Methods.

For a lens of specified NA, there are two parameters to set: the angle θ at which the lens optical axis is placed relative to an axis perpendicular to the coverslip and the angle α at which the light sheet is introduced relative to the lens optical axis (**Supplementary Fig. 13**). There are two also constraints: the lens must fit geometrically above the coverslip and the reflection of the tilted light sheet must be perpendicular to the optical axis so that it is imaged in focus. In addition to identifying appropriate parameter settings, a question arises as to what volumetric spatial resolution can be achieved for an objective lens of specified NA since views B and A' will in general not be orthogonal.

To analyze parameter settings, we start from a simple optical geometry, as shown in **Supplementary Fig. 13a**. Given the required orthogonality between the light-sheet reflection B and the objective optical axis, the angle between the optical axis of the objective and the vertical axis z must equals the angle of the introduced light sheet A relative to the mirror (here running along the lateral axis x). We denote this angle as θ. For an objective lens with half-angle less than 45 degrees, we can easily set the optical axis of the lens 45 degree relative to the mirror (i.e., θ = 45°) so that the incident light sheet A and its reflection B are orthogonal. Otherwise we tilt the objective and θ depends on the numerical aperture (NA) of the lens. If we introduce the light sheet A with angle α relative to the optical axial of the detection lens, the relationships between α and θ and the half angle of the lens can be summarized (**Supplementary Fig. 13a**):

$$\theta + \arcsin\left(\frac{\mathrm{NA}}{n}\right) = 90° \tag{33}$$

$$2\theta + \alpha = 90° \tag{34}$$

$$\theta + \alpha = \arcsin\left(\frac{\mathrm{NA}}{n}\right). \tag{35}$$

Therefore, θ and α are:

$$\theta = \begin{cases} 45° & \mathrm{NA} \le 0.707n = 0.94 \ \text{(for water immersion lens)} \\ 90° - \arcsin\left(\frac{NA}{n}\right) & \mathrm{NA} > 0.707n = \ 0.94 \ \text{(for water immmersion lens)} \end{cases} \tag{36}$$

$$\alpha = \begin{cases} 0° & \mathrm{NA} \le 0.707n \\ 90° - 2\theta = 2\arcsin\left(\frac{\mathrm{NA}}{n}\right) - 90° & \mathrm{NA} > 0.707n \end{cases}, \tag{37}$$

where n is the refractive index of immersion medium of the lens, 1.33 for water.

Assuming the objective lens has a diameter d and obeys the sine condition, the off-axis distance of the incident beam $d1$ is:

$$d1 = \begin{cases} 0 & \text{NA} \leq 0.707n \\ \dfrac{d \sin(\alpha)}{2 \sin(\alpha + \theta)} & \text{NA} > 0.707n \end{cases} . \quad (38)$$

Note that this off-axis illumination will cause a comatic aberration, but since the effective NA is small (e.g., a NA of ~0.18 for a beam waist of 1.6 μm), the magnitude of the aberration is small. In this case, the incident beam diameter $d2$ is:

$$d2 = d\frac{0.18}{\text{NA}} . \quad (39)$$

Using Equations 35-38 we find that for an NA of 1.2 in water, $\theta = {\sim}25°$, $\alpha = {\sim}39°$, $d1 = {\sim}0.35\ d$, and $d2 = {\sim}0.15\ d$.

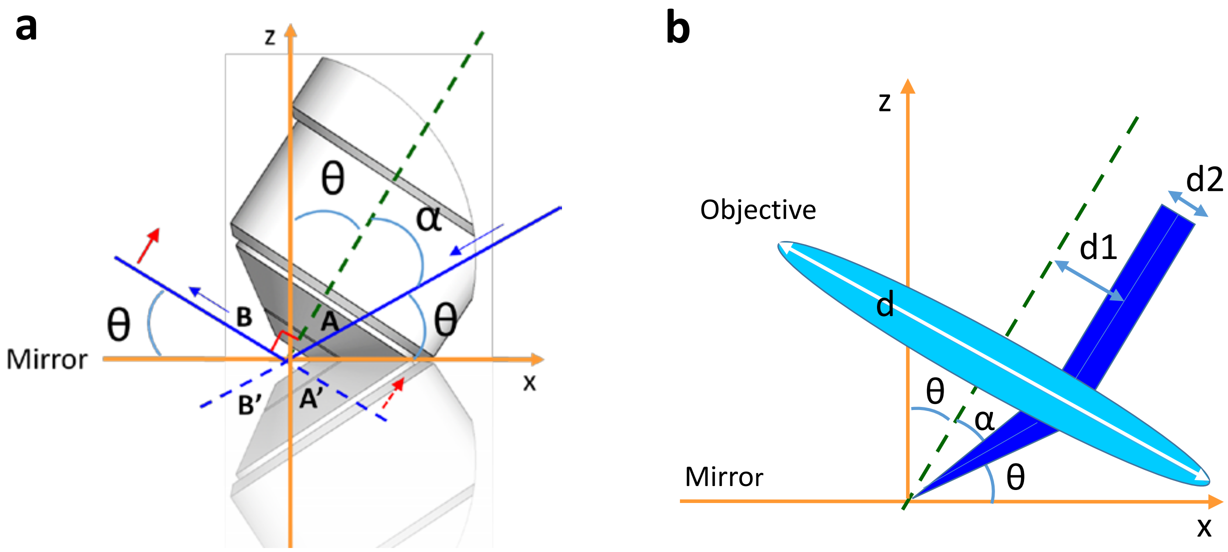

Supplementary Fig. 13, (a) *Schematic of reflective light sheet imaging using a single high NA objective for introducing excitation light sheets (blue lines) and collecting the corresponding views (red arrows). This simplified configuration allows the simultaneous acquisition of the reflected light sheet view, B, and the mirror image of the direct light sheet view, A' (red arrows), thereby improving spatiotemporal resolution and signal collection efficiency.* ***(b)*** *Higher magnification, simplified view of the objective, showing the incident angle of the tilted, low NA illumination beam.*

To estimate the spatial resolution attained in this geometry, we used simulations similar to those employed in **Supplementary Table 1** (**Online Methods)**, i.e., we simulated a point 1 μm away from the reflective coverslip, mirrored it, and blurred it according to Equation 4 in **Online Methods**; then we performed joint deconvolution according to Equations 9-10 in **Online Methods**. Below we summarize the resolution, solid angle and collection efficiencies using single-objective configurations with NAs varying from 0.8 to 1.2 (**Supplementary** Table 4). As shown, the overall collection efficiency and

volumetric resolution increases with higher NA, although the highest axial resolution (~290 nm) is achieved with NA 1.0.

Supplementary Table 4. Comparisons between reflective diSPIM imaging using single objectives with differing NA. Angle of incident light sheet relative to the coverslip, θ, is indicated in the first row.

	Reflective imaging with single 0.8 NA objective	Reflective imaging with single 0.9 NA objective	Reflective imaging with single 1.0 NA objective	Reflective imaging with single 1.1 NA objective	Reflective imaging with single 1.2 NA objective
Geometry	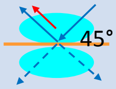	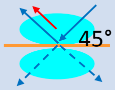	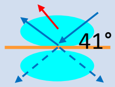	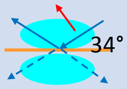	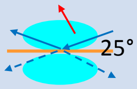
Views	Two views crossed at 90 degrees	Two views crossed at 90 degrees	Two views crossed at 98 degrees	Two views crossed at 112 degrees	Two views crossed at 130 degrees
Solid angle	0.80 π	1.05 π	1.36π	1.75 π	2.28 π
Collection efficiency	20%	26%	34%	44%	57%
Resolution from the perspective of objective (Lateral x Axial)	0.33 µm x 0.33 µm	0.30 µm x 0.30 µm	0.28 µm x 0.29 µm	0.26 µm x 0.30 µm	0.24 µm x 0.33 µm
Volumetric resolution enhancement	1x	1.33x	1.58x	1. 77x	1.89x

1. Wu, Y. *et al.* Spatially isotropic four-dimensional imaging with dual-view plane illumination microscopy. *Nat Biotechnol.* **31**, 1032-1038 (2013).
