# Supplementary material for: Reflective imaging improves spatiotemporal resolution and collection efficiency in light sheet microscopy
Source: Nat Commun. 2017 Nov 13;8:1452. doi: 10.1038/s41467-017-01250-8 (PMC5682293; doi:10.1038/s41467-017-01250-8)
Supplement: Supplementary file 3 — Description of Additional Supplementary Files [file 41467_2017_1250_MOESM3_ESM.pdf]

Description of Additional Supplementary Files

File Name: Supplementary Movie 1
Description: Raw data showing 4 views of EGFP-histone labeled *C. elegans* embryo, acquired simultaneously with the reflective diSPIM. Note pronounced epifluorescence contamination, especially in the center of the field (near the coverslip). See also Fig. 1b-c.

File Name: Supplementary Movie 2
Description: The same sample as Supplementary Movie 1, comparing 3D projections of the volume before and after deconvolution. Left: one of the 4 raw views acquired with 0.8 NA objective; Middle: naïve fusion of 4 views and resulting joint deconvolution using only spatially invariant emission point spread functions appropriate for each lens; Right: modified joint deconvolution result based on our imaging model that accounts for spatially varying background. 3D projections are rotated about the Y' axis (with respect to the coordinate system of the 0.8 NA objective). The modified model can effectively remove epifluorescence contamination and achieves isotropic resolution. See also Fig. 1b-g.

File Name: Supplementary Movie 3
Description: Time-lapse reflective imaging of a 3-fold nematode embryo expressing GCaMP3 under the *myo-3* promoter acquired with 0.8/0.8 NA diSPIM, without rolling shutter slit detection. We achieved a continuous volumetric imaging rate of 2.85 Hz (250 ms for quad view acquisition and 100 ms settling time for the piezo stage to translate back to its initial position). Maximum intensity XY and ZY projections of deconvolved reconstructions (with respect to the coordinate system of the objective) are shown, indicating rapid calcium flux within the embryo muscles. Time is indicated in seconds. See also Fig. 2.

File Name: Supplementary Movie 4
Description: Time-lapse reflective imaging of a 3-fold nematode embryo expressing GCaMP3 under the *myo-3* promoter acquired with 0.8/0.8 NA diSPIM with rolling shutter slit detection. The embryo was imaged every 600 ms, for 600 volumes. Maximum intensity XY and ZY projections of deconvolved reconstructions (with respect to the coordinate system of the objective) are shown. Time is indicated in seconds. See also Fig. 2.

File Name: Supplementary Movie 5
Description: Reflective light sheet volumetric time lapse imaging showing TurboGFP-Lck (cyan, staining the plasma membrane, endocytic machinery, and Golgi compartment) and Tom20-mApple (magenta, staining the outer mitochondrial membrane) in a U2OS cell, acquired with 0.8/0.8 NA diSPIM. XY views at three depths (0, 1.1, and 3. 7 µm depth, measured and viewed from the coverslip surface) are shown. The cell was imaged every 5 seconds over 25 minutes (400 ms to simultaneously acquire four dual-color views, and 5 seconds inter-volume pause). Time is indicated as minutes : seconds. See also Fig. 3.

File Name: Supplementary Movie 6
Description: The same sample as in Supplementary Movie 5, comparing raw and deconvolved views. Top-left: raw views in Turbo GFP-Lck channel (staining the plasma membrane, endocytic machinery, and Golgi compartment); Bottom-left: raw views in Tom20-mApple channel (staining the outer mitochondrial membrane); Top-right:

deconvolved views in Turbo GFP-Lck channel; Bottom-right: deconvolved views in Tom20-mApple channel. Maximum intensity XY projections (with respect to the coordinate system of the coverslip) are shown. See also Fig. 3.

File Name: Supplementary Movie 7
Description: Imaging volume showing Alexa Fluor 488 nm phalloidin-labeled actin in a U2OS cell plated on glass coverslips and imaged with 1.1/ 0.7 NA diSPIM. The asymmetric diSPIM system enables the visualization of fine filaments throughout the 5.2 [illegible]m-thick volume. XY views at different depths (from the perspective of the coverslip) after deconvolution are shown. See also Fig. 4f.

File Name: Supplementary Movie 8
Description: The same cell type and staining as in Supplementary Video 7, but cells were plated on a reflective coverslip. XY views at different depths (from the perspective of the coverslip) after removing epifluorescence contamination and performing joint deconvolution are shown. Images are similar to those obtained with the asymmetric system on glass coverslips. See also Fig. 4g.

File Name: Supplementary Movie 9
Description: Asymmetric reflective diSPIM imaging, showing a Jurkat T cell expressing 3XEGFP-EMTB (highlighting microtubule dynamics). Note the inward and outward displacement of microtubule bundles at base of cells, and nuclear deformation within cell interior. Four XY views at depths 0, 0.65, 1.95 and 4.16 µm from the reflective coverslip are shown. The cell was imaged every 4 seconds over 100 volumes (each volume containing two 1.1 NA views was acquired within 800 ms). Time is indicated as minutes : seconds. See also Fig. 5a.

File Name: Supplementary Movie 10
Description: Asymmetric reflective diSPIM imaging showing GFP-labeled mitochondria in U2OS cells. The movie shows the response to NaN_3 (an inhibitor of cytochrome c oxidase) added at ~13 minutes, including the fragmentation and circularization of mitochondria. Maximum intensity projections are shown on the left, and higher magnification views of yellow dashed rectangular regions are shown on the right. Cells were imaged every 15 seconds over 50 minutes (i.e., 200 volumes, and each volume containing two 1.1 NA views was acquired within 1 second). Time is indicated as minutes : seconds. See also Fig. 5b-c.

File Name: Supplementary Movie 11
Description: Asymmetric reflective diSPIM imaging showing GFP-labeled Golgi in a U2OS cell, revealing highly dynamic, ribbon-like Golgi stacks juxtaposed around the nucleus, as well as rapidly moving Golgi vesicles. Maximum intensity projections (XY and ZY views, from the perspective of reflective coverslips) are shown. The cell was imaged every 15 seconds for 45 minutes (i.e., 180 volumes, each volume containing two 1.1 NA views was acquired within 1 second). Time is indicated as minutes: seconds.

File Name: Supplementary Movie 12
Description: Asymmetric reflective diSPIM imaging showing a 3-fold nematode embryo expressing GCaMP3 from the *nmr-1* promoter, highlighting calcium transient during

backwards movement. Maximum intensity projections (XY and ZY views from the perspective of reflective coverslips) are shown. The embryo was imaged every 640 ms for 32 seconds (i.e., 50 volumes, 90 planes/volume, each volume containing two 1.1 NA views acquired within 540 seconds). Time is indicated in seconds. See also Fig. 5d-i.

File Name: Supplementary Software 1
Description: The software is compressed as a zip file. It includes four main MATLAB scripts for the deconvolution of conventional diSPIM imaging on glass coverslips (Fig. 1e, Fig. 4d-f, Supplementary Fig. 6, Supplementary Movie 7), symmetric diSPIM on reflective coverslips (Fig. 1f-g, Fig. 2, Fig. 3, Supplementary Fig. 2, Supplementary Movies 3-6), dual-view asymmetric diSPIM on reflective coverslips (Fig. 4d, Fig. 4g, Fig. 5, Supplementary Movies 8-12), and quadruple-view asymmetric diSPIM on reflective coverslips (Fig. 4g), respectively. It also includes four accessory MATLAB scripts, two for reading and writing TIFF stacks, one for performing 3D convolution in the Fourier domain, and one for computing the excitation light sheet pattern. Finally, it includes a text file that explains how to run the code.
