# Supplementary material for: Reflective imaging improves spatiotemporal resolution and collection efficiency in light sheet microscopy
Source: Nat Commun. 2017 Nov 13;8:1452. doi: 10.1038/s41467-017-01250-8 (PMC5682293; doi:10.1038/s41467-017-01250-8)
Supplement: Supplementary file 2 — Peer Review File [file 41467_2017_1250_MOESM2_ESM.pdf]

Editorial Note: This manuscript has been previously reviewed at another journal that is not operating a transparent peer review scheme. The manuscript was considered suitable for publication without further review at *Nature Communications.*
